# COVID-19 Incidence and Disease Course Among Patients at an Allergy Department

**DOI:** 10.1177/27534030231172391

**Published:** 2023-05-15

**Authors:** Louise E. van der Aa, Inge S. van Egmond, Martijn van der Sluijs, A.A. Sophie den Otter, Nadie H.M. Bosmans, Sabine E. van Beek, Angela Hartman, Niels A.D. Guchelaar, Paul L.A. van Daele, Maurits S. van Maaren, P. Martin van Hagen, Maud A.W. Hermans, Saskia M. Rombach

**Affiliations:** Department of Internal Medicine, Division of Allergy & Clinical Immunology, Erasmus University Medical Center, Rotterdam, the Netherlands; Department of Internal Medicine, Division of Allergy & Clinical Immunology, Erasmus University Medical Center, Rotterdam, the Netherlands; Laboratory of Medical Immunology, Erasmus University Medical Center, Rotterdam, the Netherlands; Department of Internal Medicine, Division of Allergy & Clinical Immunology, Erasmus University Medical Center, Rotterdam, the Netherlands; Department of Internal Medicine, Division of Allergy & Clinical Immunology, Erasmus University Medical Center, Rotterdam, the Netherlands; Laboratory of Medical Immunology, Erasmus University Medical Center, Rotterdam, the Netherlands; Department of Internal Medicine, Division of Allergy & Clinical Immunology, Erasmus University Medical Center, Rotterdam, the Netherlands

**Keywords:** COVID-19, SARS-CoV-2, allergy, hypersensitivity, incidence

## Abstract

**Background:**

Since the coronavirus pandemic in 2020, there is not much reported about the disease course of COVID-19 in patients with allergic diseases.

**Objectives:**

The aim of this study was to investigate the cumulative incidence and severity of COVID-19 among patients from the allergy department compared with the general Dutch population and people from their household.

**Design:**

We conducted a comparative longitudinal cohort study.

**Methods:**

In this study patients of the allergy department were included with their household members as a control group. Data from the beginning of the pandemic were systematically obtained through questionnaires by telephonic interviews and retrieved from electronic patient files between October 15, 2020 and January 29, 2021. Main outcomes were confirmed SARS-CoV-2 infection, disease duration, hospitalization, intensive care admission, and mortality. Questions regarding applied social distancing measures were inventoried as well.

**Results:**

Three hundred and eighty nine patients (median age 39.1 (18.7-84.7) years, 69.9% female) and 441 household members (median age 42.0 (18.0-91.5), 44.1% female) were included. The cumulative COVID-19 incidence in patients was higher compared with the general population (10.5% vs 5.6%, *P* < .001). In total, 41 (10.5%) patients attending the allergy clinic compared to 38 (8.6%) household members were infected with SARS-CoV-2 (*P* = .407). Median disease duration was 11.0 (0.0-61.0) days in patients compared to 10.5(1.0-232.0) days in household members (*P* = .996).

**Conclusion:**

The cumulative COVID-19 incidence in patients from the allergy cohort was higher compared with the general Dutch population, but similar compared with household members. There was no difference in symptoms, disease duration, or hospitalization rate between the allergy cohort and their household members.

## Introduction

Since the start of the COVID-19 pandemic, many studies tried to identify risk factors for a more severe disease course. In most cases, COVID-19 causes respiratory or gastrointestinal symptoms. However, in severe cases COVID-19 can lead to dyspnea and hypoxemia which can be seen as acute respiratory distress syndrome, causing substantial morbidity and mortality.^1^ Several comorbidities are known to increase the risk of severe illness, such as overweight, cardiovascular disease, diabetes, malignancies, chronic pulmonary disease, liver disease, kidney disease, HIV-infection or transplantation.^2–6^ However, little is known about allergic disease as a potential risk factor for severe COVID-19. In addition, literature on the relation between asthma and COVID-19 is contradictory.^7^

We hypothesize that patients from the allergy department could be more susceptible for COVID-19. Firstly, the continuous exposure to allergens in patients with inhalational allergies can lead to chronic airway and skin inflammation and thereby increased susceptibility for infections.^8,9^ Secondly, the use of systemic immunosuppressive drugs could further enhance this susceptibility, as reported in other patient cohorts using immunosuppressive drugs.^10,11^ Thirdly, SARS-CoV-2 has been shown to activate mast cells and eosinophils, which could put individuals with mast cell diseases at risk for severe COVID-19.^12^

So far, few articles with contradictory results have been published about the incidence of COVID-19 in patients with an atopic constitution. Yang et al^13^ suggested that patients with allergic rhinitis and asthma are more susceptible to COVID-19 and have more severe clinical outcomes (intensive care unit [ICU] admission, use of invasive ventilation, or death). On the other hand, Wang et al^14^ reported that allergic rhinitis does not increase the risk of COVID-19. However, these studies do not take into account a possible effect of more strictly applied social distancing measures. It is possible that patients who suffer from allergic diseases are more precautious and thus adhere to the isolation measures more strictly. Moreover, they might be more vigilant regarding self-testing because they often have chronic respiratory symptoms and/or a perceived higher risk of COVID-19.

The aim of this study was to investigate the cumulative incidence of COVID-19 in patients at the allergy department in comparison to people in their households and the general Dutch population. Hereby, the application of social distancing measures was taken into account. Secondly, the severity of the disease course among these patients was evaluated in comparison to people in their households, evaluated by the number of days experiencing symptoms, admission to the hospital, admission into the ICU and mortality.

## Methods

### Study Design and Participants

This cohort study was performed at the department of Allergy & Clinical Immunology at the Erasmus University Medical Center (Rotterdam, the Netherlands). Adult patients who attended the allergy department in the year 2020 and suffered from inhalation allergy, food allergy, medication allergy, urticaria, angioedema, or mast cell disease (mast cell activation syndrome or mastocytosis) were eligible for inclusion. The control group consisted of all household members of included patients. The main outcome measure was the cumulative incidence of a SARS-CoV-2 infection confirmed by a polymerase chain reaction (PCR) test or serology (vaccines were not yet available in 2020 in the Netherlands) in patients compared to controls. Secondary outcome measures were disease severity and adherence to social distancing measures. Disease severity was based on disease duration, hospital admission, ICU admission, and mortality, and social distancing measures included number of days going outside and number of visitors received.

### Ethical Considerations

This study (MEC-2020-0645) was approved by the local medical ethical committee and conducted according to the latest Helsinki guidelines. All participants provided informed consent.

### Data Collection

In the period between October 15, 2020 until January 29, 2021, all eligible patients were systematically contacted by phone. All patients provided informed consent before participation. Baseline characteristics, questionnaires regarding SARS-CoV-2 infection, symptoms, severity, and social distancing measures were obtained (see supplemental file 1 for the exact questionnaires). These questions were asked for 3 different periods, based on the restrictions that were advised by the government based on the number of infections in the Netherlands. The prevaccination pandemic in the Netherlands was characterized by 3 periods with high COVID-19 incidence. These periods were: the first wave (March to 1 June), with a lockdown and no test availability, the summer period (1 June to 28 September), with some restrictions and the start of test availability, and the second wave (28 September to date of interview), with a second lockdown.^15^ The same questions were answered for people in their household. Solely household members ≥18 years were included for analysis.

Comorbidities that increase a person's risk of severe illness from COVID-19 were registered. These include underlying medical conditions such as overweight, cardiovascular disease, diabetes, malignancies, chronic pulmonary disease, liver disease, kidney disease, HIV infection, or transplantation.^2–6^ Patients were asked to contact the research team if they tested positive after the interview date and were then contacted again. Between December 19, 2021 and February 14, 2022, patients with household members who tested positive were contacted again to inquire if those household members had any allergies. Allergies had to be diagnosed by a physician based on a blood or skin test (see supplemental file 2 for the questionnaire). Data from the general Dutch population were obtained through the website of the Dutch institute of health and Dutch organization for statistics (Rijksinstituut voor Volksgezondheid en Milieu and Centraal Bureau voor de Statistiek) on February 2, 2021.^16,17^

### Statistics and Data Analysis

The main outcome was the cumulative incidence of confirmed COVID-19 between October 15, 2020 and January 29, 2021. Continuous variables were presented as median (range) and categorical variables as number (%). Continuous variables were compared using the Mann-Whitney U test. Differences in groups of unpaired categorical data were analysed using a Fisher's exact test. Multivariable logistic regression analysis was performed to assess the association between the cumulative incidence of COVID-19, sex, age, comorbidity, and being a patient or household member. Multivariable lineair regression analysis was performed to assess the association between disease duration of COVID-19 and sex, age, being a patient or household member, the use of immunosuppressants and comorbidity. SPSS version 25 was used for the analyses. If an answer to a question was missing, we did not include the participant in the subanalysis for that particular question, which was also reported in the results.

Prior to statistical analysis, the adherence to social distancing was categorized according to the number of visitors received (none vs ≥ 1), number of days going outside (daily vs less than daily), type of participant (patient or household member), and COVID-19 (positive or negative). Tests were only performed for period 3 due to limited number of positive patients in periods 1 and 2. The level of significance was set at α = 0.05.

## Results

### Study Population

**Figure 1.**
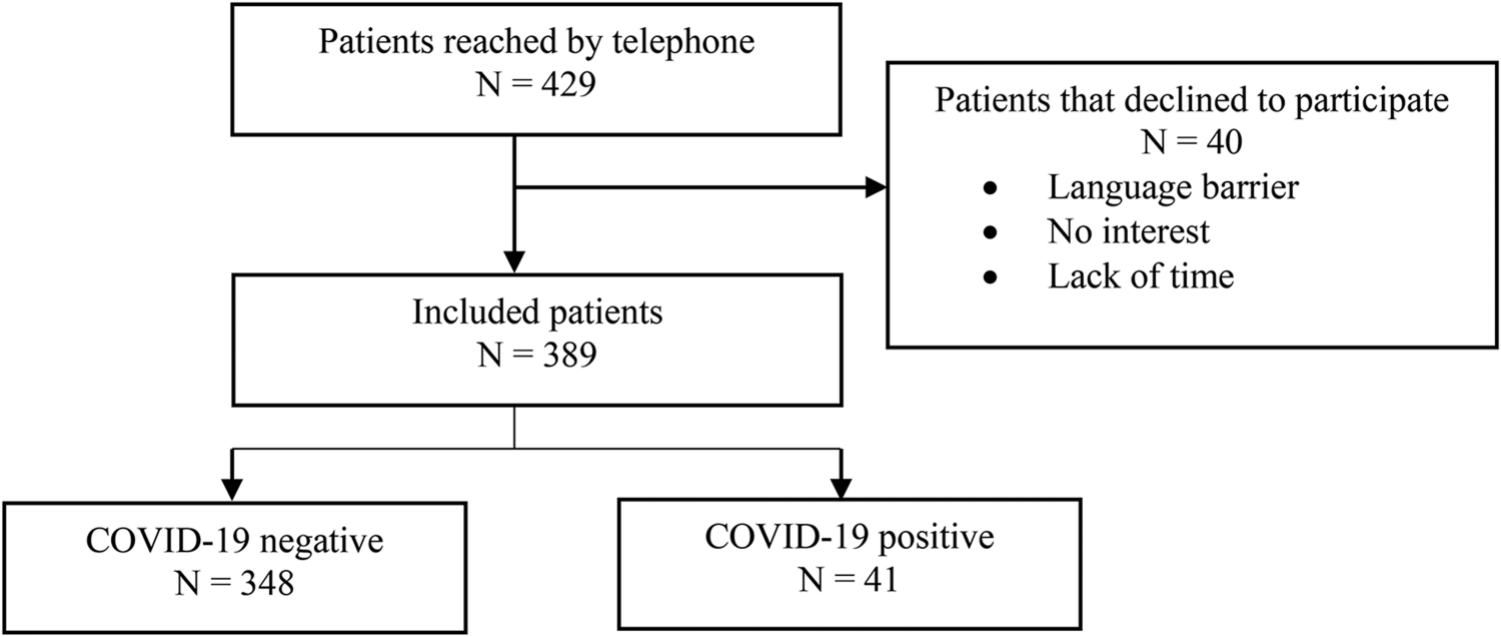
Flowchart of the inclusion process of patients from the allergy outpatient clinic.

Between October 15, 2020 and January 29, 2021, 429 patients from the allergy department were contacted for participation in the study. In total, 389 patients and 441 adult household members agreed to participate (Figure 1). Reasons for declining participation were lack of time, lack of interest, and difficulty due to language barrier. The baseline characteristics of the participants are summarized in Table 1. Patients were more often female compared with household members (69.9% vs 44.1%, respectively, *P* < .001) and had more comorbidities (58.4% vs 25.6%, respectively, *P* < .001).

**Table 1.** Clinical Characteristics of Patients in the Allergy Cohort and Their Household Members.

**Characteristics**	**Allergy, n = 389 number (%)**	**Allergy household, n = 441 number (%)**	***P* value**
**Age (years), median (range)**	39.1 (18.7-84.7)	42.0 (18.0-91.5)*	.720
**Gender: female**	272 (69.9)	194 (44.1)	<.001
**Any allergy diagnosis/urticaria/angioedema/mast cell disease**	**389** (**100)**	61/171 (35.5%) **	<.001
Airborne allergy (pollen, dust, animal dander)	236 (60.7)		
Urticaria	89 (22.8)		
Angioedema	22 (5.7)		
Bee/wasp allergy	21 (5.4)		
Medication allergy	20 (5.1)		
Food allergy	16 (4.1)		
Contact allergy	0 (0)		
Mastocytosis	13 (3.3)		
Primary mast cell activation syndrome	3 (0.8)		
**Any comorbidity**	**227** (**58.4)**	**113** (**25.6)**	**<**.**001**
Overweight (BMI > 25 kg/m^2^)	43 (11.1)	4 (0.9)	<.001
Cardiovascular disease or hypertension	45 (11.6)	23 (5.2)	.001
Diabetes	6 (1.5)	7 (1.6)	1.000
Malignancy	3 (0.8)	1 (0.2)	.346
Chronic lung disease/asthma/COPD	97 (24.9)	16 (3.6)	<.001
Kidney disease/failure	2 (0.5)	0 (0)	.219
Liver disease	2 (0.5)	0 (0)	.219
HIV	3 (0.8)	0 (0)	.103
Auto-immune disease	22 (5.7)	10 (2.3)	.018
Other comorbidities	132 (33.9)	23 (5.2)	<.001
**Any immunosuppressant use**	**233** (**59.9)**	**21** (4.8)***	**<**.**001**
Local steroids****	179 (46.0)		
Glucocorticoids	11 (2.8)		
Conventional DMARDs	4 (1.0)		
Anti-TNF	3 (0.8)		
Interleukin antagonist	4 (1.0)		
Targeted DMARDs and other biologicals	62 (15.9)		

Of note, some patients had multiple diagnoses, multiple comorbidities, and some patients used multiple immunosuppressant medication.

*The age of 1 household member was not provided (for privacy reasons).

**Allergies of household members were not specifically asked for during the first telephone call. To be sure that household members reported any allergies, the patients were e-mailed. Only a subset responded. The method of confirmation of the allergy of household members was not reported.

***For household members it was not specified what kind of immune modulating medication was used.

****Local steroids included inhaled steroids, eye drops, nasal spray, and topical steroids.

Abbreviations: BMI, body mass index; COPD, chronic obstructive pulmonary disease; DMARDs, disease-modifying antirheumatic drugs; HIV, human immunodeficiency virus; TNF, tumor necrosis factor.

Cardiovascular diseases included heart failure, cardiac valve leaks, recent myocardial infarction, hypercholesterolemia, and hypertension. Pulmonary disease included interstitial lung disease, asthma, chronic obstructive pulmonary disease and obstructive sleep apnea. Liver diseases included hepatitis, portal hypertension, Budd-Chiari syndrome, and liver cirrhosis or fibrosis, hepatoportal sclerosis and (nonalcoholic) steatosis. Auto-immune disease included rheumatoid arthritis, spondylarthritis, sarcoidosis, inflammatory bowel disease, multiple sclerosis, celiac disease, psoriasis, Hashimoto's disease, and Graves’ disease. Other comorbidities were (chronic) underlying medical conditions that were not covered by the aforementioned groups and not particularly increase the risk of severe COVID-19. The most common comorbidities in this group were eczema and thyroid dysfunction.

Glucocorticoids that were used included prednisone and hydrocortisone. Conventional disease-modifying antirheumatic drugs (DMARDs) included methotrexate, azathioprine, and cyclosporine. Antitumor necrosis factor medication that was used included adalimumab. Interleukin antagonist medication that was used were dupilumab and ustekinumab. Targeted DMARDs and other biologicals included omalizumab, mepolizumab, and dimethyl fumarate.

### Cumulative Incidence of COVID-19

The cumulative incidence of COVID-19 between the start of the pandemic in the Netherlands in 2020 and the end of January 2021 was comparable between patients and household members (10.5% vs 8.6%, *P* = .407). Between patients with airborne allergies (n = 236) and household members (n = 441), the cumulative incidence of COVID-19 was similar as well (12.7% vs 8.6%, *P* = .107). During the same time period, the cumulative incidence of COVID-19 was 5.6% in the general Dutch population.^16,17^ The cumulative incidence of COVID-19 was higher among patients from the allergy department in comparison with the general Dutch population (10.5% vs 5.6%, *P* < .001). Repeating the analysis, including patients with airborne allergies only, a higher cumulative incidence of COVID-19 was detected compared to the general population as well (12.7% vs 5.6%, *P* < .001).

The cumulative incidence for COVID-19 was neither related to being patient or household member, nor sex, nor age, nor comorbidity (Table 2).

**Table 2.** Multivariable Logistic Regression Analysis for the Cumulative Incidence of COVID-19.

**Logistic regression**	**Odds ratio (95% CI)**	***P* value**
Being patient	1.0 (0.6-1.7)	.906
Sex (male)	0.8 (0.5-1.3)	.337
Age	1.0 (1.0-1.03)	.165
Comorbidity	1.5 (0.9-2.5)	1.485

Abbreviations: CI, confidence interval; COVID-19, coronavirus disease 2019.

### Manifestation and Outcomes of COVID-19 Infection

Clinical characteristics and outcomes of patients and household members who have been infected with COVID-19 are displayed in Table 3.

**Table 3.** Clinical Characteristics and Outcomes of Patients in the Allergy Cohort and Their Household Members Infected With COVID-19.

	**Patients n = 41 (%)**	**Household members n = 38 (%)**	***P* value**
**Age (years), median (range)**	34.8 (18.8-65.6)	47.5 (19.4-65.5)	.108
**Gender: female**	33 (80.5)	17 (44.7)	.001
**Any allergy diagnosis/urticaria/angioedema/mast cell disease**	**41** (**100)**	4/32* (12.5)	**<**.**001**
Airborne allergy (pollen, dust, animal dander)	30 (73.1)	4 (12.5)	<.001
Bee/wasp allergy	0 (0)	-	
Medication allergy	1 (2.4)	-	
Food allergy	1 (2.4)	-	
Contact allergy	0 (0)	-	
Angio-oedema	3 (7.3)	-	
Chronic utricaria	11 (26.8)	-	
Mastocytosis	0 (0)	-	
Primary mast cell activation syndrome	0 (0)	-	
**Any comorbidity**	**23** (**56.1)**	**12** (**31.6)**	.**041**
Overweight (BMI > 25 kg/m^2^)	5 (12.2)	0 (0)	.056
Cardiovascular disease or hypertension	2 (4.9)	2 (5.3)	1.000
Diabetes	0 (0)	2 (5.3)	.228
Chronic lung disease/asthma/COPD	9 (22.0)	4 (10.5)	.229
Auto-immune disease	1 (2.4)	1 (2.6)	1.000
Other comorbidities	14 (33.3)	4 (10.5)	.016
**Any immunosuppressant use**	**24** (**58.5)**	**4** (**10.5)**	**<**.**001**
Local steroids only**	14 (34.1)	-	
Targeted DMARDs and other biologicals	5 (12.2)	-	
Local steroids, targeted DMARDs and other biologicals	3 (7.3)	-	
Systemic glucocorticoids only	1 (2.4)	-	
Local steroids and interleukin antagonists	1 (2.4)	-	
**COVID-19 infection**			
Period 1***	10 (24.4)	6 (15.8)	.408
Period 2***	7 (17.1)	0 (0.0)	.012
Period 3***	24 (58.5)	32 (84.2)	.014
**Symptoms**			
1 symptom	2 (4.9)	2 (5.3)	1.000
2–5 symptoms	22 (53.7)	24 (63.2)	.494
≥ 6 symptoms	17 (41.5)	12 (31.6)	.484
**Disease duration (days)**	11.0 (0.0-61.0)	10.5 (1.0-232.0)	.996
**Received COVID-19 treatment**	8 (19.5)	3 (7.9)	.196
**Admission to hospital**	0 (0)	1 (2.6)	.481
**Duration of hospital stay (days)**	NA	7	NA
**Admission to ICU**	0 (0)	1 (2.6)	.481
**Duration of ICU stay (days)**	NA	7	NA
**Mortality**	0 (0)	0 (0)	NA

Data are presented as number (percentage) or as median (range).

*Patients with COVID-19 positive household members were e-mailed and/or called to evaluate whether those household members had any allergy and in case any allergy was reported, how this was confirmed. And 32 of the total of 38 household members responded. Only allergies that were confirmed with a skin test or serology were included.

**Local steroids included inhaled steroids, eye drops, nasal spray, and topical steroids.

***Period 1: March to 1 June, period 2: 1 June to 28 September, period 3: 28 September to date of interview.

Abbreviations: BMI, body mass index; COPD, chronic obstructive pulmonary disease; COVID-19, Coronavirus Disease 2019; DMARDs;disease-modifying antirheumatic drugs; ICU, intensive care unit; NA, not applicable.

Out of 41 positive patients, 24 encountered a SARS-CoV-2 infection in the third period (September 28, 2020 up to January 2021). Thirty-seven of the 41 positive patients tested positive by PCR test and 4 had detectable antibodies after their period of active disease.

Most patients reported multiple symptoms. One patient was asymptomatic but tested positive when she was screened because a household member was infected. The most frequently mentioned symptoms among patients were fatigue (68.3%), headache (61.0%), and fever (58.5%), while household members most frequently experienced fever (57.9%), cough (57.9%), and fatigue (57.9%).

The disease duration was comparable for patients and their household members (*P* = .996, Table 3). Subgroup analysis including patients with airborne allergies only showed no difference in disease duration between patients with airborne allergies and household members (*P* = .985).

A large variation in disease duration was reported, with one outlier, reporting the longest duration of 232 days in one of the household members. The disesase duration was highly depending on how patients experienced the sequelae of COVID-19. Disease duration was not related to being patient or household member, nor sex, nor age, nor use of immunosuppressive medication, nor comorbidity (Table 4). After repeating the linear regression analysis excluding the previously mentioned outlier, disease duration was neither associated with being patient nor household member, nor sex, nor age, nor use of immunosuppressive medication nor comorbidity.

**Table 4.** Multivariable Linear Regression Analysis for Disease Duration of COVID-19 in Days.

**Linear regression**	**B-value for disease duration (95% CI)**	***P value***
Being patient	−5.3 (−20.3-9.7)	.482
Sex (male)	1.5 (−12.7-15.6)	.838
Age	−0.1 (−0.5-0.4)	.813
Use of immunosuppressants	0.7 (−15.2-16.6)	.932
Comorbidity	3.2 (−10.6-17.0)	.647

The constant in this linear regression formula was: 18.4 (−5.0 up to 41.7) days (*P* = .122).

Abbreviations: CI, confidence interval; COVID-19, Coronavirus Disease 2019.

In total 8 of the 41 (19.5%) COVID-19 positive patients of the allergy cohort received treatment. Of these patients, 4 received antibiotics, 1 received antibiotics and prednisone, 1 received prednisone, 1 received salbutamol, and 1 received prednisone and salbutamol. In total 3 household members received treatment of which 1 received antibiotics, 1 received prednisone, and 1 received remdesivir. No hospital admissions were reported among patients as opposed to 1 household member that was admitted to the ICU. This patient was a 46-year-old woman known with cardiovascular disease. No mortality was reported.

### Adherence to Social Distancing Measures

The adherence to social distancing measures is summarized in Supplemental file 3. The analysis of adherence to social distancing measures was only performed for the third period (28 September 2020 until date of interview, maximum of January 29, 2021) due to the limited number of patients that tested positive in the first and second period.

During the third period, adherence to social distancing measures was comparable between patients and household members and between COVID-19 negative and positive participants according to the questionnaires. When comparing COVID-19 positive with negative participants, COVID-19 negative participants received visitors more often (85.6% vs 68.4%, *P* < .001) and went outside on a daily basis more often (64.3% vs 50.6%, *P* = .020). The most frequently reported reasons for going outside were running errands, work, and taking a stroll. There was no difference in receiving any visitors at home or going outside on a daily basis between patients and household members. Patients without any household members were COVID-19 positive less often than patients with household members (1.5% vs 12.3%, respectively, *P* = .007).

## Discussion

In this comparative longitudinal study of a real-life cohort of patients with a variety of allergic diseases, the cumulative incidence of COVID-19 was higher in patients from the allergy department compared with the general Dutch population. However, patients did not contract COVID-19 more often than their household members and did not experience a more severe disease course.

The higher incidence of COVID-19 among patients compared with the general population is in line with a South Korean nationwide cohort study that indicated an increased likelihood of a positive Sars-CoV-2 test result in patients with asthma or allergic rhinitis.^13^ In addition, a higher incidence of COVID-19 has been reported in patients with hereditary angio-oedema without active treatment.^18^ Meanwhile, a large Chinese cohort study including Sars-CoV-2 positive patients only, showed that angiotensin-converting enzyme2 expression is not altered in patients with allergic rhinitis.^14^ Furthermore, patients with chronic urticaria are at no greater risk of contracting COVID-19 than the general population and do not develop a more severe disease course.^19,20^

It was hypothesized that a higher susceptibility of COVID-19 among patients could be caused by chronic airway inflammation due to continuous exposure to allergens.^8,9^ The majority of our patient cohort had allergic airway diseases (60.7%). However, because the cumulative incidence of COVID-19 in patients from the allergy department was comparable to their household members, an allergy by itself as a riskfactor for contracting COVID-19 is less plausible. Allergic chronic respiratory symptoms such as rhinitis resemble COVID-19. Sneezing and the need to blow the nose was even more frequent in patients with allergic rhinitis than in COVID-19 patients in a study by Bruno et al.^21^ However, in clinical practice it is difficult to distinguish COVID-19 from allergy based on symptoms alone. Thereby, these patients probably tested more often compared to people without chronic respiratory symptoms. In addition, the preparedness to test is varying in the general population, whereas patients, having appointments in the hospital, were required to have themselves tested before attending the outpatient clinic if they had contact with a COVID-19 positive patient or if they had (minor) symptoms. This could explain the higher cumulative incidence of COVID-19 in patients compared to the general Dutch population. In addition, COVID-19 could be underreported in the genereal Dutch population because of limited test availability during the beginning of the pandemic. Meanwhile, self-testing has become available in the Netherlands as an alternative for PCR testing since December 3, 2021 and as self-testing is accessible for everyone, we speculate that the test frequency is now similar in patients and the general population.^22^

As previously mentioned, patients using immunosuppressive drugs could be more susceptible to COVID-19 and have a more severe disease course.^10,11^ However, the number of patients that were treated with systemic immunosuppression in this cohort was too low to answer this question.

There was no difference in cumulative incidence nor disease severity between patients and their household members, even though these groups differed in baseline characteristics. Patients were more often female and had more comorbidities. These differences could influence the manifestation of COVID-19, as previous studies showed that male gender as well as comorbidities are associated with a more severe disease course.^3,23,24^ Therefore, an additional analysis was performed to adjust for these known risk factors, showing the cumulative incidence for COVID-19 and disease course were neither related to being patient nor household member, nor sex, nor age, nor comorbidity.

Adherence to social distancing measures in the current study did not seem to have an effect on the cumulative incidence of COVID-19, as COVID-19 negative participants received more visitors and went outside on a daily basis more often than COVID-19 positive participants. In contrast, two systematic reviews reported that adherence to public healthcare measures were associated with a lower incidence of COVID-19.^25,26^ However, these public healthcare measures were physical distancing, use of masks, eye protection, and handwashing, whereas in our cohort only the number of visitors and days of going outside were evaluated. Besides, the main reasons for going outside reported in the current study included taking a stroll or running errands, which not necessarily involve having close contact with other people. Therefore this will not substantially increase the risk of contracting COVID-19. Patients without household members were at lower risk of getting infected compared with patients that had one or more household members, suggesting that household members can bring the virus into the household.

Yet, the analysis of adherence to social distancing measures was limited to a small number of patients due to a low cumulative incidence of COVID-19 during the first and second period.

During the study period, there was no dominant variant of SARS-CoV-2 in the Netherlands, in contrast to the later dominating Alfa (B.1.1.7), Delta (B.1.617.2) and Omicron (B.1.1.529) variants.^27^ These variants might differ in incidence or disease severity, for example, the Omicron variant might have a higher transmission rate and thereby possibly a higher incidence.^28^ In addition, as the study period dates mostly from before the introduction of COVID-19 vaccines in the Netherlands and none of the participants were vaccinated yet, this study does not show the impact of vaccination.^29^ Vaccination protects up to 95% against COVID-19^30,31^ and is therefore an important factor in COVID-19 incidence and disease severity. Further research is needed to assess the effect of COVID-19 variants and COVID-19 vaccination on the incidence and disease course in patients with allergic diseases since literature on this subject is scarce.

There are several limitations in this study. Firstly, data were obtained mostly retrospectively which could lead to recall bias. Secondly, the many individual characteristics that can all influence one's course of COVID-19 remain very difficult to identify. However, the inclusion of a large cohort decreases the risk of bias due to interindividual differences. Thirdly, we only considered patients positive for COVID-19 when it was confirmed by PCR test or antibody testing. Accordingly, patients that were asymptomatic or experienced symptoms during the first wave when testing availability was limited, were not identified. However, this limitation applies for the household members as well. Fourth, because the incidence of asthma in both patients and household members was low, we could not assess the possible effect of asthma on the course of COVID-19. Therefore further studies are needed to better understand the relationship between asthma and COVID-19. At last, allergy status was not reported in all household members. Still, the number of allergic diseases reported in the household member group was lower compared to the patient group (*P* < .001), therefore it is plausible this will not change the conclusions.

Although this study was performed in an academic center, all kind of allergies, including common allergic diseases, were represented in this study.

## Conclusion

In conclusion, the cumulative incidence of COVID-19 was higher in patients from the allergy department compared with the general Dutch population (10.5% vs 5.6%, respectively, *P* < .001), but similar to their household members (8.6%). Adherence to social distancing measures including number of visitors received and number of days going outside was similar between patients and household members and did not seem to play an important factor in the risk of contracting COVID-19. However, patients without household members had a lower cumulative incidence of COVID-19 than those with household members. The disease course of COVID-19 was comparable between patients and household members.

## Supplemental Material

sj-docx-1-aar-10.1177_27534030231172391 - Supplemental material for COVID-19 Incidence and Disease Course Among Patients at an Allergy Department Click here for additional data file. Supplemental material, sj-docx-1-aar-10.1177_27534030231172391 for COVID-19 Incidence and Disease Course Among Patients at an Allergy Department by Louise E. van der Aa, Inge S. van Egmond, Martijn van der Sluijs, A.A. Sophie den Otter, Nadie H.M. Bosmans, Sabine E. van Beek, Angela Hartman, Niels A.D. Guchelaar, Paul L.A. van Daele, Maurits S. van Maaren, P. Martin van Hagen, Maud A.W. Hermans and Saskia M. Rombach in Therapeutic Advances in Allergy and Rhinology

sj-docx-2-aar-10.1177_27534030231172391 - Supplemental material for COVID-19 Incidence and Disease Course Among Patients at an Allergy Department Click here for additional data file. Supplemental material, sj-docx-2-aar-10.1177_27534030231172391 for COVID-19 Incidence and Disease Course Among Patients at an Allergy Department by Louise E. van der Aa, Inge S. van Egmond, Martijn van der Sluijs, A.A. Sophie den Otter, Nadie H.M. Bosmans, Sabine E. van Beek, Angela Hartman, Niels A.D. Guchelaar, Paul L.A. van Daele, Maurits S. van Maaren, P. Martin van Hagen, Maud A.W. Hermans and Saskia M. Rombach in Therapeutic Advances in Allergy and Rhinology

sj-docx-3-aar-10.1177_27534030231172391 - Supplemental material for COVID-19 Incidence and Disease Course Among Patients at an Allergy Department Click here for additional data file. Supplemental material, sj-docx-3-aar-10.1177_27534030231172391 for COVID-19 Incidence and Disease Course Among Patients at an Allergy Department by Louise E. van der Aa, Inge S. van Egmond, Martijn van der Sluijs, A.A. Sophie den Otter, Nadie H.M. Bosmans, Sabine E. van Beek, Angela Hartman, Niels A.D. Guchelaar, Paul L.A. van Daele, Maurits S. van Maaren, P. Martin van Hagen, Maud A.W. Hermans and Saskia M. Rombach in Therapeutic Advances in Allergy and Rhinology

## Declarations

**Ethics Approval and Consent to Participate:** The local medical ethical committee (Medical Ethical Review Committee of the Erasmus Medical Center) approved this study (MEC-2020-0645). Spoken informed consent was provided by all participants and the study was conducted according to the latest Helsinki guidelines.

**Availability of Data and Materials:** Available on request.
