# Supplementary material for: COVID-19 Incidence and Disease Course Among Patients at an Allergy Department
Source: Ther Adv Allergy Rhinol. 2023 May 15;14:27534030231172391. doi: 10.1177/27534030231172391 (PMC10189845; doi:10.1177/27534030231172391)
Supplement: sj-docx-1-aar-10.1177_27534030231172391 - Supplemental material for COVID-19 Incidence and Disease Course Among Patients at an Allergy Department [file sj-docx-1-aar-10.1177_27534030231172391.docx]

# Supplementary file 1: Questionnaire COVID-19 for patients from the allergy department

1. What is your date of birth?
2. What is your diagnosis?
3. What kind of immune suppression do you use (multiple answers possible, name of the medication)?
4. Did you experience any COVID-19 related symptoms since 28 February 2020 (date of first registered COVID-19 infection in the Netherlands)? (0=none 1=fever 2 = nasal cold 3= sore throat 4= dyspnoea 5= cough 6= chest pain 7=anosmia 8= headache 9=diarrhoea 10= muscle and/or joint pain 11= skin abnormalities 22=fatigue)
5. Did you have physical contact with anyone infected with COVID-19?
6. Were you tested for COVID-19? If so, where were you tested? (0=not tested , 1=GGD, 2= general practitioner 3= hospital 4=work, 5= other, …)
7. How many times were you tested?
8. What was the result of the test?
9. What was the test date?
10. Has there been found another cause for your complaints?

**Questions 11-20 only have to be answered if the patient tested positive for COVID-19.*

1. On which date did your symptoms begin?
2. What kind of symptoms did you have during your infection? (0=none 1=fever 2 = nasal cold 3= sore throat 4= dyspnoea 5= cough 6= chest pain 7=anosmia 8= headache 9=diarrhoea 10= muscle and/or joint pain 11= skin abnormalities 22=fatigue)
3. In case of fever, what was your highest temperature measured?
4. On which date did your symptoms end?
5. How many days did you experience symptoms in total?
6. Did you get any treatment for your infection? If so, what kind of treatment? (0= none 1=immunosuppression was discontinued 2=antibiotics 3= remdesivir 4=tocilizumab, 5=dexamethasone 6= other medication, …)
7. Were you admitted to the hospital (general ward)?
8. If so, how many days were you admitted to the general ward?
9. Were you admitted to the ICU?
10. If so, how many days were you admitted to the ICU?
11. From march till the 1^st^ of June: did you apply the social distancing measures as advised by the government?
12. From march till the 1^st^ of June: did you still receive visitors? (1=yes, more than 6 people at a time, 2= yes, 3-6 people at a time, 3=yes, less than 3 people at a time. 4=none)
13. From march till 1^st^ of June: How many times a week did you leave the house? (1= never, 2= once or twice a week, 3= three till six times a week, 4= daily)
14. From the 1^st^ of June till the 28^th^ of September: did you apply the social distancing measures as advised by the government?
15. From the 1^st^ of June till the 28^th^ of September: did you still receive visitors? (1=yes, more than 6 people at a time, 2= yes, 3-6 people at a time, 3=yes, less than 3 people at a time. 4=none)
16. From the 1^st^ of June till the 28^th^ of September: How many times a week did you leave the house? (1= never, 2= once or twice a week, 3= three till six times a week, 4= daily)
17. From the 28^th^ of September till the date of the interview: did you apply the social distancing measures as advised by the government?
18. From the 28^th^ of September till the date of the interview: did you still receive visitors? (1=yes, more than 6 people at a time, 2= yes, 3-6 people at a time, 3=yes, less than 3 people at a time. 4=none)
19. From the 28^th^ of September till the date of the interview: How many times a week did you leave the house? (1= never, 2= once or twice a week, 3= three till six times a week, 4= daily)
20. For what reason do you leave the house? (1= groceries, 2= work, 3= social events, 4= informal care, 5= medical, 6=other reasons)
21. How many people are in your household?

*The following questions should be answered about every person in the household separately. They can be answered by the patient.*

1. What is the relation of this household member to the patient?
2. What is the age of this household member? (preferably date of birth, otherwise age or birth year)
3. What is the sex of this household member?
4. Does he or she have any comorbidities? (1= overweight, 2= cardiovascular diseases/ hypertension, 3= diabetes, 4= malignancy, 5= kidney disease/ failure, 6= chronic lung disease/asthma/COPD 7= liver disease 8= HIV-infection 9=transplantation 10=auto-immune diseases 11= other, …)
5. Does he or she use immunosuppression?
6. Did he or she experience any COVID-19 related symptoms since 28 February 2020 (date of first registered COVID-19 infection in the Netherlands)? (0=none 1=fever 2 = nasal cold 3= sore throat 4= dyspnoea 5= cough 6= chest pain 7=anosmia 8= headache 9=diarrhoea 10= muscle and/or joint pain 11= skin abnormalities 22=fatigue)
7. Was he or she tested for their symptoms?
8. What was the result of the test?

**Questions 9-22 only have to be answered if the member of the household tested positive for COVID-19.*

1. In case of fever, what was the highest temperature measured?
2. Did he or she have physical contact with anyone infected with COVID-19?
3. Where did he/she test for COVID/19? (0=not tested , 1=GGD, 2= general practitioner 3= hospital 4=work, 5= other, …)
4. How many times was he/she tested?
5. What was the result of the test?
6. What was the test date?
7. What was the first day of his/her symptoms?
8. What was the last day of his/ her symptoms?
9. How many days did he/she experience symptoms in total?
10. Did he/she get any treatment for his/her infection? If so, what kind of treatment? (0= none 1=immunosuppression was discontinued 2=antibiotics 3= remdesivir 4=tocilizumab, 5=dexamethasone 6= other medication, …)
11. Was he/she admitted to the hospital (general ward)?
12. If so, how many days was he/she admitted to the general ward?
13. Was he/she admitted to the ICU?
14. If so, how many days was he/she admitted to the ICU?
15. From march till the 1^st^ of June: did you apply the social distancing measures as advised by the government?
16. From march till the 1^st^ of June: did you still receive visitors? (1=yes, more than 6 people at a time, 2= yes, 3-6 people at a time, 3=yes, less than 3 people at a time. 4=none)
17. From march till 1^st^ of June: How many times a week did he/she leave the house? (1= never, 2= once or twice a week, 3= three till six times a week, 4= daily)
18. From the 1^st^ of June till the 28^th^ of September: did he/she apply the social distancing measures as advised by the government?
19. From the 1^st^ of June till the 28^th^ of September: did he/she still receive visitors? (1=yes, more than 6 people at a time, 2= yes, 3-6 people at a time, 3=yes, less than 3 people at a time. 4=none)
20. From the 1^st^ of June till the 28^th^ of September: How many times a week did he/she leave the house? (1= never, 2= once or twice a week, 3= three till six times a week, 4= daily)
21. From the 28^th^ of September till the date of the interview: did he/she apply the social distancing measures as advised by the government?
22. From the 28^th^ of September till the date of the interview: did he/she still receive visitors? (1=yes, more than 6 people at a time, 2= yes, 3-6 people at a time, 3=yes, less than 3 people at a time. 4=none)
