# Supplementary material for: COVID-19 Incidence and Disease Course Among Patients at an Allergy Department
Source: Ther Adv Allergy Rhinol. 2023 May 15;14:27534030231172391. doi: 10.1177/27534030231172391 (PMC10189845; doi:10.1177/27534030231172391)
Supplement: sj-docx-2-aar-10.1177_27534030231172391 - Supplemental material for COVID-19 Incidence and Disease Course Among Patients at an Allergy Department [file sj-docx-2-aar-10.1177_27534030231172391.docx]

# Supplementary file 2: Questionnaire allergies for COVID-positive household members

*The following questions should be answered about every person in the household separately. They can be answered by the patient.*

1. Does he/she have any allergies?
2. Which allergies does he/she have?
3. How was this allergy diagnosed? (bloodtest, skintest, other)
4. By whom was this allergy diagnosed? (himself/herself, general practitioner, internist, other physysian)
