# Supplementary material for: COVID-19 Incidence and Disease Course Among Patients at an Allergy Department
Source: Ther Adv Allergy Rhinol. 2023 May 15;14:27534030231172391. doi: 10.1177/27534030231172391 (PMC10189845; doi:10.1177/27534030231172391)
Supplement: sj-docx-3-aar-10.1177_27534030231172391 - Supplemental material for COVID-19 Incidence and Disease Course Among Patients at an Allergy Department [file sj-docx-3-aar-10.1177_27534030231172391.docx]

# Supplementary file 3: Adherence to social distancing measures by patients and household members

| **Allergy cohort (n=389) (%)** | | | | **Household members (n=441) (%)** | | | | |  |
| --- | --- | --- | --- | --- | --- | --- | --- | --- | --- |
| **Social distancing measures** | **Period 1** | **Period 2** | **Period 3** | | **Social distancing measures** | **Period 1** | **Period 2** | **Period 3** | |
| **Adherence** | 383(98.5) | 382(98.2) | 383(98.5) | | **Adherence** | 434(98.4) | 427(96.8) | 427(96.8) | |
| **Number of visitors received** |  |  |  | | **Number of visitors received** |  |  |  | |
| No visitors | 80(20.6) | 45(11.6) | 64(16.5) | | no visitors | 75(17.0) | 46(10.4) | 69(15.6) | |
| 1-3 people | 291(74.8) | 226(58.1) | 305(78.4) | | 1-3 people | 340(77.1) | 267(60.5) | 341(77.3) | |
| 3-6 people | 18(4.6) | 111(28.5) | 20(5.1) | | 3-6 people | 25(5.7) | 120(27.2) | 31(7.0) | |
| >6 people | 0(0) | 7(1.8) | 0(0) | | >6 people | 1(0.2) | 8(1.8) | 0(0) | |
| **Number of days outside per week** |  |  |  | | **Number of days outside per week** |  |  |  | |
| Never | 8(2.1) | 1(0.3) | 4(1.0) | | never | 3(0.7) | 1(0.2) | 0(0) | |
| 1-2 days | 21(5.4) | 14(3.6) | 14(3.6) | | 1-2 days | 10(2.3) | 9(2.0) | 10(2.3) | |
| 3-6 days | 115(29.6) | 109(28.0) | 118(30.3) | | 3-6 days | 168(38.1) | 153(34.7) | 161(36.5) | |
| Daily | 245(63.0) | 265(68.1) | 253(65.0) | | Daily | 260(59.0) | 278(63.0) | 270(61.2) | |

Period 1 was from March 2020 until 1^st^ of June 2020, period 2 was from 1^st^ of June 2020 until 28^th^ of September 2020 and period 3 was from 28^th^ of September until January 2021.
